# Microbial and Qualitative Traits of Quinoa and Amaranth Seeds from Experimental Fields in Southern Italy

**DOI:** 10.3390/foods12091866

**Published:** 2023-04-30

**Authors:** Anna Reale, Maria Cristina Messia, Cataldo Pulvento, Antonella Lavini, Stefania Nazzaro, Tiziana Di Renzo

**Affiliations:** 1 Institute of Food Sciences, National Research Council (CNR-ISA), Via Roma 64, 83100 Avellino, Italy; 2 Department of Agricultural, Environmental and Food Sciences (DiAAA), University of Molise, Via De Sanctis, 86100 Campobasso, Italy; 3 Department of Soil, Plant and Food Science (DISSPA), University of Bari “A. Moro”, Via Amendola, 165/A, 70126 Bari, Italy; 4 Institute for Agricultural and Forestry Systems in the Mediterranean (ISAFOM), National Research Council of Italy (CNR), 80055 Portici, Italy

**Keywords:** pseudocereals, spore-forming bacteria, *Bacillus* spp., PCR-DGGE, rope spoilage, bread

## Abstract

Quinoa and amaranth are of special interest since they are increasingly used for the development of new bakery products with enhanced nutritional value. The aim of the study was to evaluate the agronomic, microbiological, and nutritional characteristics of quinoa and amaranth seeds grown in Southern Italy. For this reason, quinoa Titicaca and three amaranth accessions (5, 12, and 14) were cultivated in different experimental fields in the Campania Region and analyzed for the cultivation aspects, chemical composition, and microbiological quality of the seeds. All seeds showed a good adaptability to cultivation in the experimental areas of the Mediterranean basin. Quinoa seeds were characterized by their higher protein, fat, and ash content than the amaranth seeds, which were characterized by their higher value in dietary fiber. All seeds, regardless of the geographical area of production, were contaminated with yeasts, moulds, and spore-forming bacteria, mainly *Bacillus cereus*, *B. licheniformis*, *B. safensis* and *B. subtilis*, as identified by 16S rRNA sequencing analysis. So, the detection of *Bacillus* spp. must be strongly monitored, as quinoa and amaranth seeds could be used in bread production, where they can cause ropiness, resulting in great economic losses for the industries.

## 1. Introduction

In the last years, the growing demand for healthier and functional diets has led to an increase in the consumption of quinoa and amaranth seeds, which possess interesting nutritional characteristics. These seeds are widely used for their high gluten-free protein content, high amount of nutraceutical compounds, and nutritionally balanced amino acid composition [1,2,3]. Quinoa and amaranth are both dicotyledonous plants, classified as pseudocereals and native to the South America, which preferentially grow under different pedo-climatic conditions, and both crops can withstand numerous abiotic factors, such as salinity and dryness [4,5]. Amaranth seed yields recorded in Europe ranged from 1200 to 6700 kg/ ha [6], and quinoa tested in Italy under field conditions showed seed yields between 1 and 4 t/ha [5]. Their ability to withstand severe weather conditions makes them excellent crops to grow in countries where global climate change is most extreme. Thanks to their nutritional and agronomic characteristics, the cultivation of quinoa and amaranth has spread outside their areas of origin in recent decades. Different kinds of research have been carried out to study the adaptability of quinoa and amaranth in Mediterranean areas increasingly affected by abiotic stress due to climate change [6,7,8,9,10,11,12]. For this reason, they are also recognized as promising crops to fight hunger and achieve food security worldwide. Nowadays, quinoa and amaranth seeds are used in numerous food preparations to develop new functional and technologically innovative food products. The lack of the gluten fraction makes these seeds suitable for the production of dietary foods recommended for people with a gluten allergy [13].

Quinoa and amaranth seeds are often used as ingredients in salads, soups, and smoothies, and their flours are also used in the baking industry for the production of flakes, snacks, crackers, tortillas, and biscuits [14,15,16]. In the last years, many commercial gluten-free breads are incorporating pseudocereal flour or seeds up to 20–30% in order to improve the quality of the final product [17,18,19]. Meanwhile, quinoa and amaranth breads, due to their high water activity and nutritional richness, are a favorable substrate for microbial growth, especially of filamentous fungi and bacteria [20]. Among the main agents responsible for the bread spoilage, known as ropiness, are *Bacillus* species such as *B. subtilis*, *B. licheniformis*, *B. amyloliquefaciens*, *B. pumilus*, and *B. cereus*. This alteration is characterized by discoloration of breadcrumbs and the occurrence of fruit odor. Breadcrumbs become soft and sticky, and they can almost liquefy over time [21]. For these reasons, monitoring the presence of the *Bacillus* genus in raw materials destined for the baking industry assumes considerable importance. Members of the genus *Bacillus* are ubiquitous bacteria with a wide distribution in food and the environment, and they are generally considered harmless contaminants [22]. The genus includes several Gram-positive rods able to produce endospores when environmental conditions are adverse to growth. Endospores are highly resistant to different treatments during food production, so they can persist in industrial plants and processed foods [23]. The spores can germinate if foods are not properly refrigerated, causing food poisoning in consumers. Therefore, monitoring the occurrence of *Bacillus* genus in raw materials used for the baking industry is necessary mainly in warm climates of Mediterranean countries, where hot and humid conditions persist.

For these reasons, the aim of this article was to evaluate (a) the adaptability of quinoa and amaranth cultivars to grow under field conditions in the Mediterranean basin of Southern Italy; (b) the nutritional and qualitative characteristics of the seeds obtained under the different agronomic conditions; and (c) the main microbiological characteristics, with particular regard to *Bacillus* spp. occurrence, of quinoa and amaranth seeds collected in different experimental fields. These evaluations of quinoa and amaranth seeds are of particular interest because they could be considered as alternative crops suitable for typical Mediterranean climatic conditions. In addition, the nutritional and microbiological characterisation of the seeds provided useful scientific indications for the potential development of new bakery products with high nutritional value.

## 2. Materials and Methods

### 2.1. Materials and Reagents

Chemicals and reagents used in the study were of analytical grade and were obtained from Sigma-Aldrich chemistry (Madrid, Spain). Media for microbiological analysis were from Oxoid (Milan, Italy).

### 2.2. Experimental Field and Samples

The study was conducted on a Danish cultivar of quinoa with a short cycle length (Titicaca) and three amaranth accessions (5, 12 and 14) from the University of Copenhagen (Department of Agriculture and Ecology Faculty of Life Sciences); both quinoa varieties and amaranth accessions were selected for European condition.

Seeds of quinoa and amaranth analyzed in the study came from different experimental fields placed in the Campania Region, as described below. The field trials were carried out in the 2013–2014 season by personnel from CNR-ISAFoM (National Research Council—Institute for Agricultural and Forest Mediterranean Systems).

Three experimental quinoa fields have been carried out for quinoa in different areas of the Campania Region: (a) CNR-ISAFoM research station located in Vitulazio (CE); (b) CNR-ISAFoM Insitute in Ercolano (NA); and (c) Stiscia farm located in Montecalvo Irpino (AV).

The three different amaranth accessions were instead cultivated at the CNR-ISAFoM research station in Vitulazio (CE).

Table 1 shows the specifications of the experimental locations, the quinoa genotype and amaranth accessions, the type of soil, environmental climates, and altitude ranges of the sites.

All the trials were carried out under rain conditions; plots were disposed in a randomized complete block design with three repetitions and using a theoretical density of 200,000 plants/ha. Planting, weeding, harvesting, and seed cleaning operations were carried out manually.

**Figure 1:**
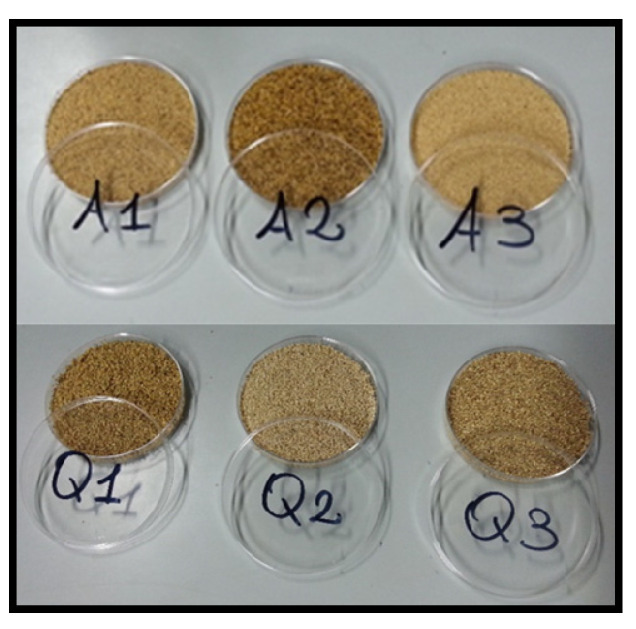
Amaranth seeds (A1, A2 and A3) and Quinoa (Q1, Q2 and Q3) seeds grown in the Mediterranean basin of Southern Italy object of experimentation.

Quinoa and amaranth seeds from different fields (Figure 1) were collected immediately after harvest and were stored at 4 °C until physico-chemical and microbiological analyses were performed. Figure S1 illustrates the experimental design of the research.

### 2.3. Biomass, Yield and Seed Chemical Composition Analysis

At physiological maturity, the plants were manually harvested and then threshed using a stationary threshing machine (Plot 2375, Cicoria srl, Palazzo San Gervasio, Italy) [7]. The seed yield, 1000-seed weight, and above-ground biomass (AGB) were determined and the harvest index (HI) was calculated as the ratio of yield to total AGB.

Prior chemical analyses of amaranth and quinoa seeds from each harvest were ground using a refrigerated laboratory mill (model IKA A10-IKAWERKE; GmbH &CO. KG, Staufen, Germany).

Moisture, ash, and fat content were determined according to ICC methods 109/1, 104/1, and 136, respectively [24]. The dietary fiber was determined according to AACC method 32.05 [25]. Protein content was determined through a Leco nitrogen determiner (model FP 528 Leco Corp., St. Joseph, MI, USA) according to the Dumas combustion method, AACC method 46-30.01 [25] (Nx6.25). Total starch was quantified enzymatically by a Megazyme assay kit (K-TSTA, Megazyme International Ltd., Bray, Ireland).

The water activity (Aw) was determined at 20 °C by using the Rotronic HP23 HygroPalm model, following the manufacturer’s instructions, as reported previously [26].

The pH was measured on 10 g of seed samples after homogenization in 90 mL distilled water for 2 min in a Stomacher laboratory blender (BAG MIXER 400, Interscience, Saint-Nom-la-Bretèche, France) with a Medidor PH Basic 20 pHmeter (CRISON, Alella, Spain) [3].

### 2.4. Color Measurement

The color was measured using CIE (Commission Internationale de l’Eclairage) L*, a* and b* color system, where L* describes brightness, a* is redness, and b* is yellowness. Color measurements were performed in triplicate with a colorimeter (model CR300 Minolta Italia, S.p.A., Milan, Italy).

### 2.5. Microbiological Analysis

For microbial analyses, 10 g of each product was aseptically transferred into a sterile stomacher bag and diluted with 90 mL of physiological solution (9 g/L NaCl). After 1 min shaking in a Stomacher apparatus (BAG MIXER 400, Interscience, France), the samples were serially diluted and plated. Total mesophilic bacteria were determined on Plate Count Agar after incubation at 28 °C (FALC instruments SRL, Treviglio, Italy) for 48 h. *Enterobacteriaceae* were estimated on VRBGA after 36 h incubation at 37 °C (FALC instruments SRL, Treviglio, Italy). Total and faecal coliforms were counted on VRBA after 36 h incubation at 37 °C and 44 °C (FALC instruments SRL, Treviglio, Italy), respectively. Enterococci were counted on Slanetz and Bartley medium after 36 h incubation at 37 °C. Yeasts and moulds were quantified on YPD agar plates (bacteriological peptone 20 g/L, dextrose 20 g/L, yeast extract 10 g/L, agar 20 g/L, and 4 mg/100 mL streptomycin). Counts were performed after 48–72 h incubation at 28 °C. Lactic acid bacteria (LAB) were counted on De Man, Rogosa, and Sharpe (MRS) agar, and on 4 mg/100 mL cycloheximide (SIGMA Aldrich, Schnelldorf, Germany) after incubation at 28 °C for 72 h under anaerobic conditions (Gas Pack AnaeroGen TM, OXOID). The results of viable counts were expressed as a log of colony forming units per gram of seeds (Log cfu/g).

### 2.6. Bacillus *spp.* Isolation and Phenotypic Characteristics

Spore-forming bacteria were isolated, as reported previously [22]. Briefly, 20 g of each sample was diluted with 180 g of a sterile Bacto-peptone (Difco, Detroit, MI, USA) solution (0.1%, *w*/*v*) and homogenized in a Stomacher for 2 min. The suspension was filtered through sterile Whatman paper No 4 (Whatman, Maidstone, UK) and heat treated for 20 min at 90 °C to select spores. The suspension was then poured into plates (1 mL), decimally diluted, and spread on Starch Agar (SA, Difco) (100 μL). The plates were incubated for 24 h at 30 °C, and the number of presumptive *Bacillus* was counted and expressed as CFU/g seeds. For each sample, 7–12 colonies randomly picked from SA plates were purified by streaking on fresh medium and incubated as described previously. The purified isolates were stored on slant at 4 °C for further characterization. Gram staining, catalase test, microscopic observation, cell motility, and presence of endospores were used to screen the isolates and to presumptively identify those belonging to the genus *Bacillus*.

### 2.7. Molecular Identification

*Bacillus* spp. was identified by PCR-DGGE analysis. Briefly, for the first PCR process, the genomic DNA of strains was amplified using the primers BacF (5′-GGGAAACCGGGGCTAATACCGGAT-3′) [27] and R1378 (5′-CGGTGTGTACAAGGCCCGGGAACG-3′) [28]. The 25 μL PCR reaction mixture included 1 μL of bacterial DNA, 0.15 μM of each primer, 1 × PCR reaction buffer (Biotechrabbit, Berlin, Germany), 0.2 mM of dNTP mixture, 2.5 U of Taq polymerase (Biotechrabbit, Germany), 25 mM of MgCl_2_, and topped up with sterile distilled water. PCR was performed in a Nexus Mastercycler (Eppendorf, Hamburg, Germany) using the following amplification conditions: one cycle at 94 °C for 5 min, 35 cycles at 94 °C for 1 min, 65 °C for 90 s, and 72 °C for 2 min followed by an additional cycle of 10 min at 72 °C. Subsequently, this initial PCR product was diluted 1:100 and used as a template for a second PCR with primers F968 [29] and R1378 [28]. A GC clamp (5′-CGCCCGGGGCGCGCCCCGGGCGGGGCGGGGGCACGGGGGG-3′) was added to the forward primer (F968) according to Araùjo and others [29]. The program used for the second PCR was as follows: initial denaturation at 94 °C for 5 min; two cycles of 94 °C for 1 min, 63 °C for 1 min, 72 °C for 2 min, followed by 10 times the same cycle with every second one at 2 °C lower annealing temperature (until 55 °C); and 20 cycles of 94 °C for 1 min, 55 °C for 1 min, and 72 °C for 2 min, followed by the final extension at 72 °C for 10 min.

Negative control without a DNA template was included. The initial PCR product and the nested PCR product were purified and stored at 4 °C. The PCR products were electrophoresed on 1.5% agarose for size and quality.

### 2.8. DGGE Analysis and Sequencing

The amplicons obtained were subjected to DGGE analysis, using a DCode Universal Mutation Detection System (BioRad, Hercules, CA, USA). Electrophoresis was performed in a 0.8-mm thick polyacrylamide gel (8% [*w*/*v*] acrylamide-bisacrylamide [37.5:1]) with a denaturant gradient from 40% to 60% (100% denaturant corresponds to 7 M urea and 40% [*w*/*v*] formamide) increasing in the direction of the electrophoresis run (120 V, 60 °C, 5 h). Gels were stained for 30 min in 1.25 × TAE containing Gel Red Nucleic Acid Stain 3x (Biotium, Hayward, CA, USA) and visualized under UV illumination. DGGE gels were digitally acquired by GEL DOC XR System (Bio-Rad, Hercules, CA, USA) using the software Quantity One Analysis (Bio-Rad) and analyzed with the pattern analysis software package, Gel Compare II v.6.6 software (Applied Maths, Sint-Martens-Latem, Belgium). The calculation of similarities in the profiles of the bands was based on Pearson product–moment correlation coefficient. The dendrogram was obtained through the Unweighted Pair Group Method using the Arithmetic Average (UPGMA) clustering algorithm.

A total of one to five representative strains of each cluster obtained by DGGE analysis were amplified using the same primer pairs without the GC clamp, as previously described. The amplified ones were purified using the QIAquick PCR purification kit (QIAGEN GmbH, Hilden), sent for sequencing (Eurofins Genomics, Ebersberg, Germany), and sequence homology and identification were then carried out as reported previously [30].

The identified *Bacillus* strains were stored, as frozen stocks, at −80 °C (50%, *w*/*v* in glycerol) in the Microbial Culture Collection of the Institute of Food Sciences—National Research Council (ISA-CNR; Avellino, Italy).

### 2.9. Statistical Analysis

Analysis of variance (ANOVA) as a Randomised Complete Block design with three replications was carried out on agronomic data, and the means were compared using the Fisher’s least significant difference (LSD) test at a 5% significance level.

The physico-chemical and microbiological analyses were carried out in triplicate. Mean values and standard deviation were calculated. Analysis of variance was performed to determine significant differences (Tuckey’s HSD test *p < 0.05) between means.

## 3. Results and Discussion

Table 2 shows the results for the grain yield, above-ground biomass (AGB) harvest index (HI), and 1000-seed weight of quinoa and amaranth seeds produced in the different experimental fields. The amaranth accessions (samples A1, A2 and A3) had seed yields ranging between 1.34 and 2.20 t/ha. In particular, sample A1 showed significantly lower seed yield and biomass values than samples A2 and A3, while the HI value and the 1000 seed weight were not significantly different among the amaranth samples analyzed. The data confirmed the good adaptability of grain amaranth under Southern Italian pedo-climatic conditions, as reported in previous studies [31,32].

Regarding the samples of quinoa Titicaca (Q1, Q2 and Q3), the seed yield values recorded in the three different experimental areas varied significantly; the highest seed yields (2.3 t/ha) were recorded in the Ercolano area (Q1) and the lowest (0.19 t/ha) in the Montecalvo Irpino area (Q3). The low seed yield performances recorded in Montecalvo Irpino (Q3) were due to the different climatic conditions of the hilly windy area such as lower air temperatures. The lowest harvest index (9%) was also recorded in the Montecalvo Irpino area, due to the adverse climatic conditions. In fact, during the crop period, a high average evapotranspiration demand was found in the hilly area due to strong winds, which led to a greater growth of the stem diameter and a reduced leaf expansion (data not reported). Seed yield data of quinoa Titicaca registered in the Vitulazio and Ercolano areas were in agreement with the data from the literature, confirming the wide environmental adaptation of this crop [11,33]. Amaranth accession 12 and 14 (A2, A3) recorded significantly higher seed yields and above-ground biomass respect accession 5 (A1); no significant differences were recoded for amaranth HI values between the three accessions. The 1000 seed weight showed no significant differences among both amaranth and quinoa samples grown in different areas, indicating that this parameter is not really influenced by different pedo-climatic conditions.

The nutritional (g/100 g d.w.) and physical properties of quinoa and amaranth seeds are given in Table 3.

Starch is the main component of both amaranth and quinoa seeds, and was present in amounts between 49% and 59% dry matter; obtained data were in agreement with previous studies carried out on the Titicaca variety [34,35] and amaranth seeds [36], and were consistent with the composition data of gluten free flour reported by Hager et al. [37].

The fat content of quinoa seeds ranged between 5.9% and 6.6% d.m., as reported in other studies [35,38,39], and it was almost double that of amaranth seeds. This is attributable to the high proportional size of the embryo within the quinoa seeds, which makes quinoa one of the pseudocereals with the highest fat content [37].

Contrary to what has been reported by De Bock et al. [40], quinoa seeds of this experimentation showed a protein content on average higher than that of amaranth (mean value 15.6%d.w. and 13.9% d.w., respectively). The high interest in these pseudocereals, expressed by several parties (food industries, research institutions), is related to both the absence of gluten proteins and the high quality of the protein component rich in essential amino acids, which improves the biological value of proteins [3].

The significantly lower weight of 1000 seeds in amaranth seeds (Table 2) justifies the higher fiber content (Table 3) compared to quinoa seeds due to the smaller size of the seeds and, thus, a higher incidence of the cortical layers rich in fiber.

However, the differences found in chemical composition, as reported in the literature [37], can be attributed not only to the specific characteristics of each species and variety but also to both the pedo-climatic conditions of the areas where the cultivation took place and the agronomic practices adopted.

Table 3 also shows the pH and Aw values of the seeds. In particular, the pH values of the amaranth seeds of the different samples were very similar to each other and ranged between 5.36 ± 0.20 (sample A3) and 5.43 ± 0.09 (sample A2). Quinoa samples also did not differ significantly in pH values, recording values between 5.39 ± 0.15 (sample Q1) and 5.62 ± 0.17 (sample Q3).

Overall, the pH value of seeds can be influenced by the specific characteristics of the varieties but, above all, to the pedo-climatic conditions of the areas where the cultivation took place and the agronomic practices adopted for the experiments (chemical characteristics of the soil, adoption of different fertilization, and rainfall occurrence). The seed samples recorded very low Aw values, ranging from 0.427 ± 0.024 (sample Q3) to 0.514 ± 0.024 (sample A3). Our results are similar to those reported by other authors for amaranth, chia, and sesame seeds [41].

The low Aw of seeds is a characteristic of non-perishable foods, and is one of the most important parameters for the preservation of dry products [41].

The color parameters of quinoa and amaranth seeds are presented in Table 4.

Color analysis of the seed samples highlighted some differences. The amaranth seeds differed mainly in brightness, (L*); in particular, sample A2 was darker than A1 and A3, with the latter having the highest L* value. Small differences were noted for parameters “a” and “b” for the amaranth samples. For quinoa seeds, sample Q2 was lighter than the others and was characterized by less redness (a*) and more yellowness (b*) than Q2 and Q3. As pointed out by other authors, the color variation of quinoa and amaranth seeds depends primarily on phenotypic variability [42,43]. Furthermore, environmental conditions also play a very important role in color determination. Indeed, Granado-Rodriguez et al. [44] studied the color of Titicaca quinoa seeds over three years of cultivation in Spain. These authors reported higher values of L* (50.3–56.3), a* (5.5–6.8) and b* (20.8–24.2) parameters than our results on the same variety of quinoa seeds.

**Figure 2:**
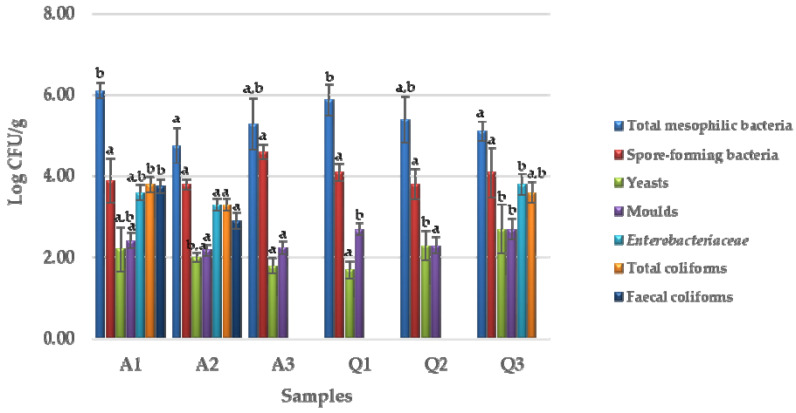
Viable counts of the amaranth and quinoa seeds. Means ± standard deviations of triplicate independent experiments are shown. Letters on plot bars indicate significant differences (*p* < 0.05) in viable count for each microbial group within the different seeds (a, b).

Figure 2 shows the viable counts (Log cfu/g) of the microbial groups analyzed in the amaranth and quinoa seed samples.

The samples had total mesophilic loads between 4.75 ± 0.43 Log cfu/g (sample A2) and 6.10 ± 0.19 Log cfu/g (A1). Yeasts and moulds were detected in all samples with microbial loads between 1.7 ± 0.21 Log cfu/g (Q1) to 2.7 ± 0.61 Log cfu/g (Q3) and 2.18 ± 0.15 Log cfu/g (A2) and 2.7 ± 0.15 Log cfu/g (Q1 and Q3), respectively. Spore-forming bacteria were found in all the samples in high concentration between 3.8 ± 0.37 Log cfu/g (A2 and Q2) and 4.6 ± 0.18 Log cfu/g (A3). Enterococci and lactic acid bacteria (LAB) had load values < 1 Log cfu/g; instead, *Enterobacteriaceae* and total coliforms were only found in the sample A2 (about 3 Log cfu/g), A1, and Q3 (about 4 Log cfu/g). Faecal coliforms were only found in samples A1 (3.76 ± 0.17 Log cfu/g) and A2 (2.90 ± 0.19 Log cfu/g). Yeasts and moulds were found in all the samples in the same concentrations.

Microbiological contamination of cereal and pseudocereals seeds derives from several sources and occurs during plant growth, harvest, post-harvest drying, and storage [45]. Paz et al. [46] isolated from quinoa different Gram positive bacteria such as *Bacillus* spp. and *Staphylococcus epidermidis* and Gram negative bacteria such as *Enterobacter*, *Salmonella, Escherichia,* and *Klebsiella*. Furthermore, Noelting et al. [47] isolated from amaranth seeds different fungal genera such as *Alternaria*, *Aspergillus*, and *Fusarium*. In our samples, the microbial groups found consisted mainly of yeasts, moulds, and spore-forming bacteria.

Other authors also found yeasts and moulds with similar microbial load in different seeds from Mexico and Portugal [41]. Spore-forming bacteria were also found in spices from the United States (with values from 2.0 ×10^2^ to 8.3 ×10^7^ CFU/g) [48] and in Australian wheat (10^4^ CFU/g) [49].

The flour is considered a microbiologically safe product since microbial growth is inhibited at low Aw. However, more than 10^3^ spores/g can be found in flours, which remain dormant for a long period of time, affecting bread quality and shelf life. Spores can survive during food processing treatments and germinate when proper conditions are restored, causing food poisoning in consumers [50].

**Figure 3:**
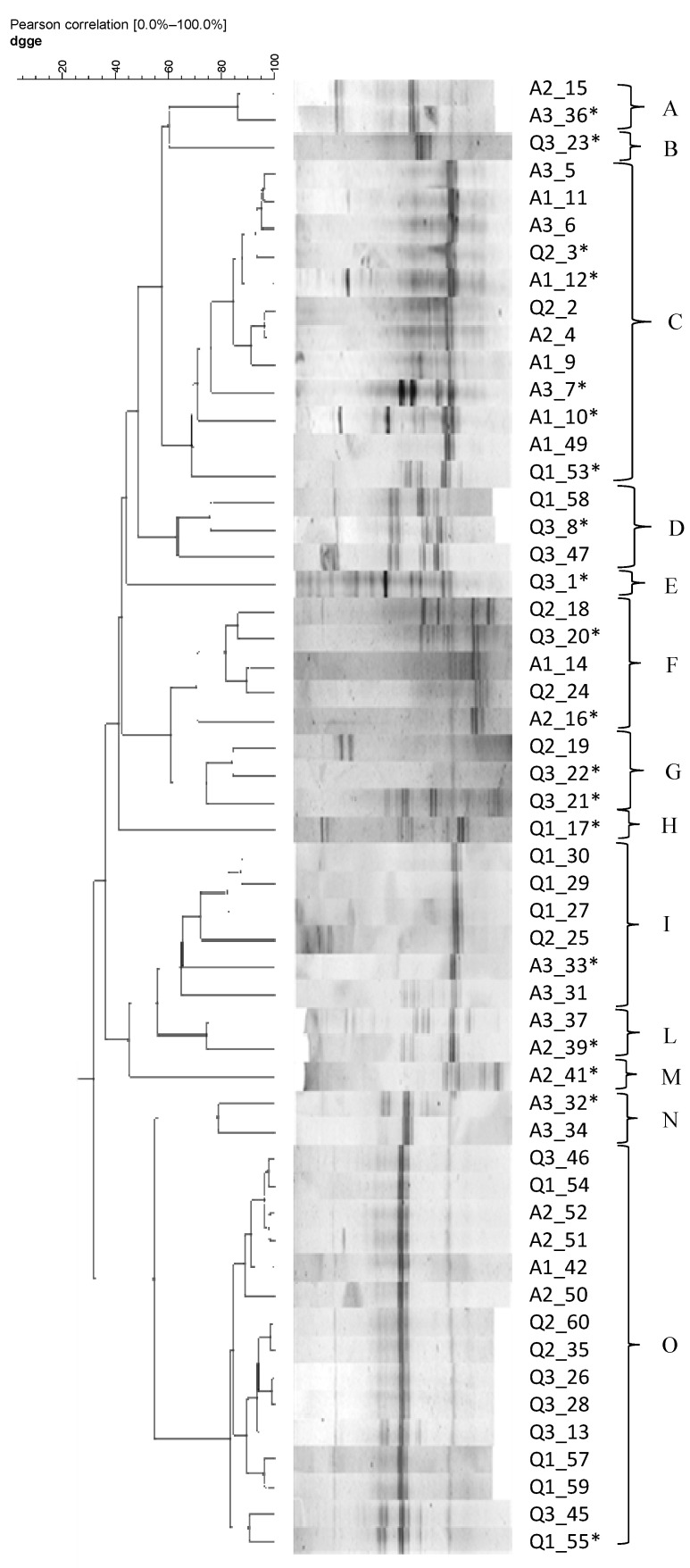
Dendrogram showing the similarity among DGGE profiles of DNA extracted from spore-forming bacteria isolated from quinoa and amaranth seeds. Asterisks (*) indicate those isolates identified by sequencing.

Spore-forming bacteria detected in the quinoa and amaranth samples were isolated and subjected to genetic identification. Thus, 60 colonies (10 from each sample) were isolated from the starch agar plates and purified for molecular identification. Of the 60 isolates, 54 were Gram-positive, rod-shaped and positive in sporulation and catalase tests, were presumptively identified as belonging to the genus *Bacillus* and subjected to PCR-DGGE identification. PCR-DGGE and 16S rRNA sequencing were used to identify the species. DGGE analysis resulted in the dendrogram shown in Figure 3.

Combining these results with those obtained from DGGE cluster analysis, it was possible to identify 47 of the 54 isolated strains. Only seven strains, grouped in clusters A, B, D, and H, were not identified as they resulted from uncultured bacterium cloned by sequencing analysis (see Table 5) (Figure 3). The identified isolates were found to belong to the genus *Bacillus*. In detail, 21 strains (about 47% of the isolated strains) were identified as *B. subtilis*, 17 strains (about 36% of the isolated strains) as *B. cereus*, 5 as *B. safensis*, and 4 as *B. licheniformis*. The last two species were isolated with a frequency of ≤10%.

Other authors have also found a high frequency of the genus *Bacillus* in quinoa seeds. Members of the genus *Bacillus* are ubiquitous bacteria [51], with a wide distribution in food and the environment and are generally considered harmless contaminants. *Bacillus* spp. are among the main food spoilage organisms due to their versatile metabolism and heat-resistant spores that are also highly resistant to radiation, desiccation, and chemical agents [52]. Given the increasing use of quinoa and amaranth seeds for the production of special breads with improved health characteristics, the assessment of their microbiological quality is particularly important, since spoilage microorganisms, mainly *Bacillus* spp., can cause large economic losses for industries, and, for this reason, the monitoring of these species should be routinely done in raw materials used for bread making.

Pitzschke [53] found that white cultivar real seeds, harvested in Bolivia, are contaminated by *Bacillus* spp. potentially used for application in the agriculture, food, and cosmetics industries. Castillo et al. [54] found that quinoa hosts many cultivable fungi and bacteria, among which the genus *Bacillus* predominates. *Bacillus* was also found in other low-moisture foods such as flours, spices [55], and various starchy foods such as potatoes and unhusked rice [56].

In our study, regardless of the geographical area of production, quinoa and amaranth seeds harbor a large presence of *Bacillus* with a fair biodiversity, as four different species were found. The presence of *Bacillus*, in quinoa and amaranth seeds, may be caused by contamination during growth, harvesting, and subsequent storage processes.

The species *Bacillus cereus* and *Bacillus subtilis* were found in all the samples analyzed (A1, A2, A3, Q1, Q2 and Q3). These species were found in cereals and cereal derivatives, as also reported in other studies [57,58,59].

*Bacillus licheniformis*, on the other hand, was found only in two quinoa samples (Q2 and Q3) and in a sample of amaranth (A2). *B. subtilis* and *B. licheniformis* were frequently detected in raw materials used in bread making and can cause ropy spoilage of bread [23,60]. Rope spoilage is considered a re-emerging spoilage phenomenon characterized by sticky crumbs, discoloration, slime formation, and fruity odor [61] due to the action of proteolytic and amylolytic enzymes of *Bacillus* spp. [22,50]. Ropiness is not only an economic problem for industries but also a health risk, as several *Bacillus* species can cause food poisoning through their toxins. *Bacillus cereus*, for instance, produces the heat-stable toxin cereulide and at least three enterotoxins [50], and the presence of *B. subtilis* and *B. licheniformis* at levels ranging from 10^5^ to 10^9^ CFU/g has been associated with food-borne illness [62].

The presence of *B. safensis* was also found in almost all seed samples analyzed, with the exception of the sample A3 of amaranth and the quinoa sample (Q1). *B. safensis*, isolated for the first time from spacecraft surfaces [63], was more recently isolated from condensed milk, surface soil, plants, and oil fields [64,65,66]. It is considered a plant growth-promoting bacterium and has promising biotechnological applications due to its ability to produce different enzymes and secondary metabolites [67], with antifungal, antibacterial, and cytotoxic activities [68,69]. In addition, it can be considered an industrially safe bacterium as no study to date has highlighted its pathogenicity.

## 4. Conclusions

Based on the data collected in the present study, all the objectives of the work were fulfilled. 

(a) The cultivation of quinoa Titicaca and amaranth (accessions 5, 12, and 14) can be considered a viable alternative to be included in the cropping systems of Southern Italy for their good adaptability to grow under field conditions in the Mediterranean basin. In the best experimental field conditions evaluated, seed yields of more than 2 t/ha were recorded.

(b) Chemical analysis revealed a different nutritional composition of the pseudocereals analyzed. Protein, fat, and ash content was higher in quinoa samples, whereas dietary fiber values were higher in amaranth seeds. Therefore, both pseudocereals can be used to improve the nutritional aspects of food products.

(c) Regardless of the geographical area of production, quinoa and amaranth seeds harbored a large presence of food spoilage microorganisms such as yeasts, moulds and *Bacillus* spp. In detail, the occurrence of three different *Bacillus* species such as *B. licheniformis, B. cereus*, and *B. subtilis*, responsible for bread spoilages, was ascertained. These findings are of particular importance for bakery industry, especially, where the presence of these microorganisms can produce significant economic losses.

## Figures and Tables

**Table 1:** Location and description of the experiment agro-climatic sites of the pseudocereal samples under study.

Sample		Type of Pseudocereal	Location	Geographic Position	Altitude (m.a.s.l.)	Soil Type	Average Annual Rainfall (mm)
**AMARANTH**	**A1**	Amaranth accession 5	Vitulazio (CE)	14°50′ E, 40°07′ N	25	clay loam	805
	**A2**	Amaranth accession 12	Vitulazio (CE)	14°50′ E, 40°07′ N	25	clay loam	805
	**A3**	Amaranth accession 14	Vitulazio (CE)	14°50′ E, 40°07′ N	25	clay loam	805
**QUINOA**	**Q1**	Quinoa var. Titicaca	Ercolano (NA)	14°21′ E, 40°50′ N	175	sandy	1080
	**Q2**	Quinoa var. Titicaca	Vitulazio (CE)	14°50′ E, 40°07′ N	25	clay loam	805
	**Q3**	Quinoa var. Titicaca	Montecalvo Irpino (AV)	15°3′ E, 41°15′ N	518	clay loam	1068

**Table 2:** Grain yield, above-ground biomass (AGB), harvest index (HI) and thousand seeds weight of amaranth and quinoa samples.

Sample	Seed Yield	Biomass	Harvest Index	1000 Seeds Weight
	t/ha		%	g
**A1**	1.34 ^b^	23.70 ^b^	6	0.95
**A2**	2.20 ^a^	28.90 ^a^	8	0.68
**A3**	1.96 ^a^	28.4 ^a^	7	0.95
*p*-value	<0.05	<0.05	n.s	n.s
**Q1**	2.3 ^a^	6.7 ^a^	34 ^a^	2.45
**Q2**	1.33 ^b^	5.85 ^ab^	23 ^b^	3.00
**Q3**	0.19 ^c^	2.10 ^b^	9 ^c^	2.97
*p*-value	<0.05	<0.05	<0.05	n.s

Values followed by different letters are significantly different among treatments according to LSD test at *p*  <  0.05; n.s. means non-significant.

**Table 3:** Chemical composition (g/100 g d.w.), water activity and pH of amaranth and quinoa seeds.

Sample		Protein	Lipid	Ash	Total Starch	Dietary Fiber	Water Activity (a_w_)	pH
**AMARANTH**	**A1**	13.7 ± 0.02 ^a^	4.0 ± 0.05 ^b^	2.0 ± 0.11 ^a^	54.3 ± 0.55 ^b^	15.1 ± 1.00 ^a^	0.468 ± 0.022 ^a^	5.42 ± 0.11 ^a^
	**A2**	14.4 ± 0.15 ^b^	3.2 ± 0.03 ^a^	3.2 ± 0.13 ^c^	48.9 ± 0.61 ^a^	19.1 ± 1.23 ^b^	0.496 ± 0.020 ^a^	5.43 ± 0.09 ^a^
	**A3**	13.7 ± 0.01 ^a^	3.3 ± 0.09 ^a^	2.4 ± 0.20 ^b^	56.0 ± 0.43 ^c^	13.5 ± 0.98 ^a^	0.514 ± 0.024 ^a^	5.36 ± 0.20 ^a^
**QUINOA**	**Q1**	16.5 ± 0.09 ^c^	5.9 ± 0.06 ^a^	6.0 ± 0.11 ^b^	54.9 ± 0.69 ^a^	10.8 ± 0.94 ^a^	0.436 ± 0.014 ^a^	5.39 ± 0.15 ^a^
	**Q2**	15.9 ± 0.12 ^a^	6.5 ± 0.09 ^b^	5.3 ± 0.23 ^a^	58.7 ± 0.75 ^c^	10.2 ± 0.85 ^a^	0.445 ± 0.012 ^a^	5.47 ± 0.08 ^a^
	**Q3**	14.3 ± 0.04 ^b^	6.6 ± 0.10 ^b^	5.6 ± 0.24 ^a^	56.8 ± 0.73 ^b^	10.6 ± 0.70 ^a^	0.427 ± 0.024 ^a^	5.62 ± 0.17 ^a^

Means ± standard deviations of triplicate independent experiments are shown.Within each column, for quinoa and amaranth samples, overall means with different superscript letters are significantly different (*p* < 0.05).

**Table 4:** Color characteristics of amaranth and quinoa seeds.

Sample		Colorimetric Indices		
		L*	a*	b*
**AMARANTH**	**A1**	48.02 ± 0.39 ^b^	5.99 ± 0.07 ^a^	20.71 ± 0.32 ^b^
	**A2**	38.93 ± 0.58 ^a^	5.97 ± 0.05 ^a^	19.55 ± 0.40 ^a^
	**A3**	50.04 ± 0.26 ^c^	5.66 ± 0.03 ^b^	21.13 ± 0.14 ^b^
**QUINOA**	**Q1**	44.14 ± 0.16 ^a^	4.92 ± 0.02 ^c^	18.46 ± 0.07 ^b^
	**Q2**	46.74 ± 0.17 ^b^	4.24 ± 0.05 ^a^	18.87 ± 0.13 ^c^
	**Q3**	44.74 ± 0.63 ^a^	4.58 ± 0.14 ^b^	17.39 ± 0.25 ^a^

Means ± standard deviations of triplicate independent experiments are shown. Within each column, for quinoa and amaranth samples, overall means with different superscript letters are significantly different (*p* < 0.05).

**Table 5:** Identification, based on blast comparison in GenBank, of 19 strains selected on the basis of DGGE cluster analysis.

Cluster	Strain	Size (bp)	Closest Relative	% Identity	Source ^a^
A	A3_36	1363	Uncultured bacterium clone	100	KF066707
B	Q3_23	1372	Uncultured bacterium clone	97	GQ017951
C	Q2_3	1462	*B. subtilis*	99	JN942155
C	A1_12	1414	*B. subtilis*	99	KF830999
C	A3_7	878	*B. subtilis*	99	KP699115
C	A1_10	1417	*B. subtilis*	99	KT720106
C	Q1_53	1416	*B. subtilis*	99	KP347686
D	Q3_8	919	Uncultured bacterium clone	99	JX283599
E	Q3_1	1417	*B. subtilis*	99	KT720106
F	Q3_20	940	*B. safensis*	99	HM583998
F	A2_16	878	*B. safensis*	99	HM583998
G	Q3_21	1002	*B. licheniformis*	99	KR782291
G	Q3_22	1395	*B. licheniformis*	97	KT318805
H	Q1_17	1464	Uncultured bacterium clone	99	JQ940780
I	A3_33	1417	*B. subtilis*	99	KT720106
L	A2_39	1401	*B. subtilis*	99	KM458977
M	A2_41	1395	*B. licheniformis*	99	KT318805
N	A3_32	1504	*B. cereus*	99	GU056810
O	Q1_55	734	*B. cereus*	99	DQ339658

^a ^ Accession number of the sequence of the closest relative found by blast search.

## Data Availability

Data presented in this study are available on request from the corresponding author.

## Supplementary Materials

The following supporting information can be downloaded at: https://www.mdpi.com/article/10.3390/foods12091866/s1. Figure S1: Experimental design.
