# Supplementary figures and images for: Microbial and Qualitative Traits of Quinoa and Amaranth Seeds from Experimental Fields in Southern Italy
Source: Foods. 2023 Apr 30;12(9):1866. doi: 10.3390/foods12091866 (PMC10177794; doi:10.3390/foods12091866)

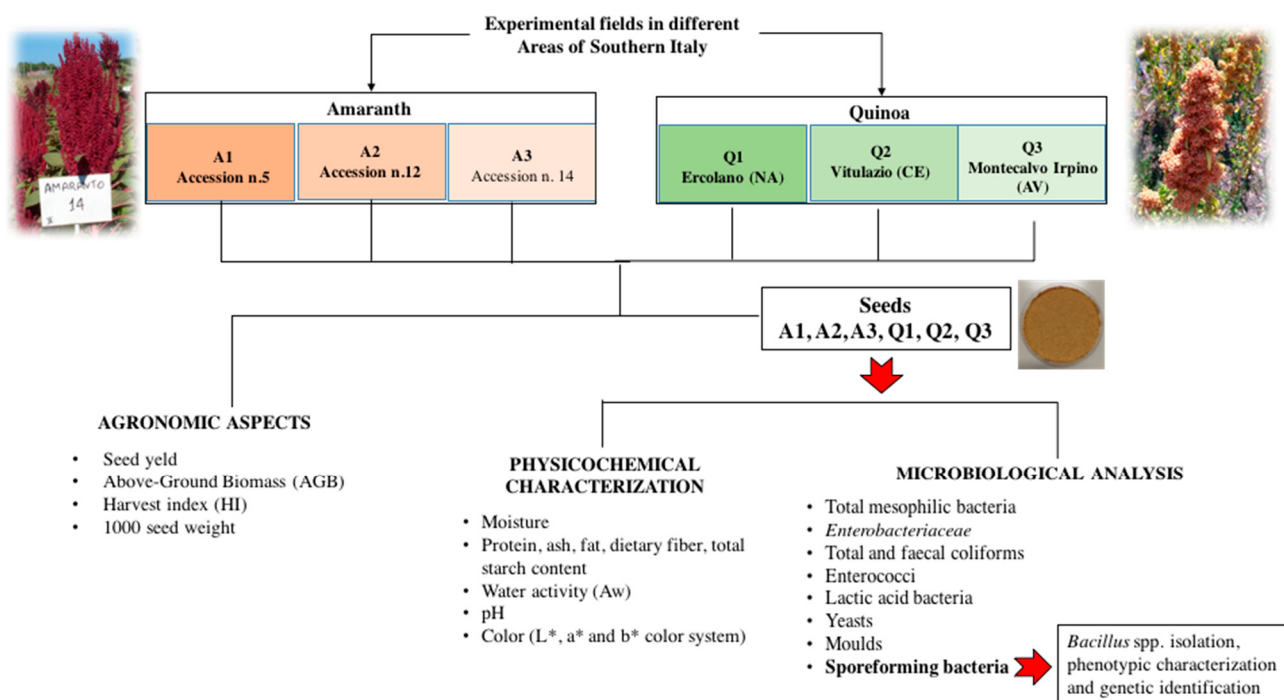

**Figure S1: Experimental design**

Supplement: Supplementary file 1 [file foods-12-01866-s001.zip › foods-2360463-supplementary.pdf]
